# Correction of technical bias in clinical microarray data improves concordance with known biological information

**DOI:** 10.1186/gb-2008-9-2-r26

**Published:** 2008-02-04

**Authors:** Aron C Eklund, Zoltan Szallasi

**Affiliations:** 1 Children's Hospital Informatics Program at the Harvard-MIT Division of Health Sciences and Technology (CHIP@HST), Harvard Medical School, Boston, MA 02115, USA; 2 Center for Biological Sequence Analysis, Department of Systems Biology, Technical University of Denmark, DK-2800 Lyngby, Denmark

## Abstract

The performance of gene expression microarrays has been well characterized using controlled reference samples, but the performance on clinical samples remains less clear. We identified sources of technical bias affecting many genes in concert, thus causing spurious correlations in clinical data sets and false associations between genes and clinical variables. We developed a method to correct for technical bias in clinical microarray data, which increased concordance with known biological relationships in multiple data sets.

## Abstract 2

A method is presented to correct for technical bias in clinical microarray data, which increases concordance with known biological relationships.

## Background

The introduction of massively parallel measurement techniques such as gene expression microarrays has led to a much disputed paradigm shift in experimental design [1]. When a single biochemical entity is quantified it is customary and expected to include replicates and carefully selected controls such as calibration curves, which increase the confidence in the validity of the measurements. However, due to the relatively high expense of microarrays and the scarcity of starting material, many microarray data sets feature only a single measurement per sample. Beyond financial pressure the main justification for this approach has been the notion that the overall behavior of a large number or all probes on a given gene chip provides a reliable estimate of the systematic measurement bias associated with any given probe on the microarray. For example, the widely used microarray normalization methods are based on the assumption that if the average probe intensity tends to be brighter on a given chip than on others, then the estimated expression value of any given probe will be overestimated; therefore, individual probe intensities on the brighter chip have to be adjusted accordingly, bringing the average intensity in line with other chips.

Controlled reference data sets featuring spike-ins or mixtures have demonstrated the possibility of high accuracy in measurements and have been valuable for comparison of normalization algorithms and analytical techniques [2-4]. However, it is unclear whether these data sets are representative of real-world clinical data, or whether an algorithm that performs well on these data sets also performs well on clinical data.

## Results and discussion

In most clinical microarray data sets, very little is known about the true expression levels, and without such reference points it is difficult to evaluate the effectiveness of data transformations or statistical methods. However, one possible criterion is that multiple samples taken from the same tumor should be more similar to each other than they are to samples taken from other tumors. In a breast cancer data set previously published by Signoretti *et al*. [5], 98 surgical specimens were profiled on Affymetrix HG-U95Av2 arrays. Of these, eighteen samples form nine replicate pairs, in which two samples were taken from adjacent sections of the same frozen block [5]. After normalizing these data with the robust multi-array average (RMA) algorithm [6], we performed hierarchical clustering and found that only four of the nine replicate pairs clustered together (Figure 1a).

**Figure 1:**
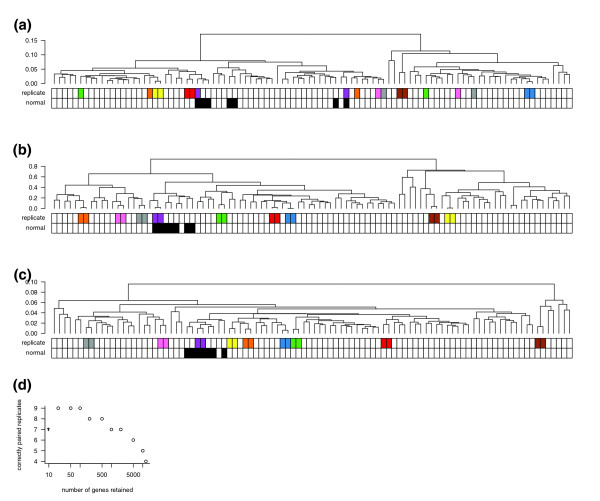
Bias correction improves concordance of replicates in a large breast cancer microarray study. Hierarchical clustering was performed, and the number of replicate samples correctly paired (joined at the lowest node) was used as a measure of concordance. Colors underneath the dendrograms indicate replicate samples in which each pair was taken from a single tumor specimen, and black squares indicate samples taken from normal breast. **(a) **The unfiltered, uncorrected data results in only four of nine replicate pairs together. **(b) **Filtering out all but the top 100 genes with highest variance results in all 9 pairs clustered together. **(c) **Bias correction as described in this paper also results in all nine replicate pairs clustered together, but in this case all genes are retained. **(d) **The concordance achieved by variance filtering is sensitive to the number of genes retained. Generally, filtering out low-variance probe sets with increasing stringency leads to increased concordance between replicate sample pairs.

In an effort to increase the number of replicate pairs clustering together, we tried various thresholds for filtering out low-variance genes. This common practice is intended to reduce noise by removing genes that are essentially unchanged between samples [7]. As expected, we found that using increasingly stringent thresholds yielded more replicate pairs clustered together (Figure 1d). When we removed all but the most variable 100 genes, each of the 9 replicate pairs clustered together (Figure 1b). Satisfyingly, seven normal (non-tumor) samples also clustered together, which had not occurred with the unfiltered data. Thus, at least in this data set, strong filtering of expression data results in dendrograms that more accurately reflect known relationships between samples. Such filtering might be appropriate if the goal is to extract robust clinical markers. However, for other types of analysis (for example, when seeking information about a predetermined set of genes with known biological function) such extreme filtering may eliminate valuable information, in which case correction of noisy data would be preferable to its removal.

Although it is remotely possible that the dendrogram resulting from the original, unfiltered data (Figure 1a) indicates biologically relevant relationships, we assumed that the disruption of replicate pairs is an artifact caused by noise in the filtered-out genes. If this noise were entirely random, filtering out genes would be the only way to reduce noise. However, if the noise were systematically biased, it might be possible to characterize the bias and correct the measured expression values. Notably, even a small amount of systematic bias could have substantial influence on clustering, if the bias affects a large enough subset of genes in a coordinated fashion.

Therefore, we searched for evidence of systematic bias in the Signoretti data set. We anticipated that any systematic bias would affect a subset of genes having some common characteristic, rather than a random subset of genes. Such bias would then tend to increase the apparent correlation between pairs of genes within this subset. By visualizing the correlation between pairs of probe sets, as a function of intensity, we observed intensity-dependent correlation bias (Figure 2a). Remarkably, this intensity-dependent correlation bias was present in all data sets we checked [8-12] and was not improved by replacing RMA normalization with Microarray Suite 5 (MAS5), model-based expression index (MBEI) [13], or GeneChip robust multi-array average (GCRMA) normalization [14] (Additional data file 1). Importantly, the bias was essentially undetectable after post-summary quantile normalization (PSQN), a secondary normalization step that gives each sample the same distribution of expression values, demonstrating that the bias is related to the distribution of intensities and does not reflect biologically relevant gene relationships (Additional data file 1). Also, PSQN applied to the unfiltered Signoretti data set increased the number of paired replicates from four (out of nine) to five (data not shown).

**Figure 2:**
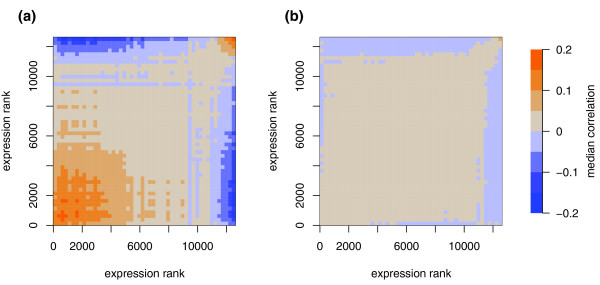
Spurious intensity-dependent correlations between pairs of genes and between genes and bias metrics. Probe sets were sorted by mean expression and divided into 50 bins of approximately 250 probe sets each. For each possible pair of bins, the median correlation coefficient between probe sets is indicated by color. Thus, the presence of colored regions indicates intensity-dependent correlation bias between pairs of probe sets. **(a) **In the uncorrected data, the correlation between a given pair of probe sets is biased by the mean expression value of each probe set. For example, two probe sets that are both highly expressed on average are more likely to be positively correlated than expected by chance. **(b) **In the bias-corrected data, the correlation bias is reduced.

Because PSQN only partially resolved the misclustering of replicates in the Signoretti data, we sought to understand the underlying causes of the bias. Two sources of variation, those arising during hybridization and those intrinsic to the starting RNA, seem especially likely to influence many probe sets in a coordinated manner. During hybridization, a subset of probes is susceptible to nonlinear effects caused by saturation at high intensities or by the noise floor at low intensities [15]. For these probes, changes in target concentration have a diminished effect on probe intensity compared to probes in the linear regime. Thus, if samples are loaded onto arrays at slightly different concentrations, or are hybridized or washed under slightly different conditions, any subsequent normalization is likely to overcorrect probes in the nonlinear range and undercorrect those in the linear range. The use of multiple probes and robust normalization schemes may reduce the effect of these nonlinear probes, but our observation of intensity-dependent bias (Figure 2a) indicates that many normalization schemes that do not account for systematic bias may be inadequate in some cases.

A second potential source of bias is the variable quality and quantity of starting RNA, which may be especially problematic in data sets featuring samples from surgical specimens [16]. For an individual transcript to contribute to the measured signal, it must be intact between the region targeted by microarray probes and the poly-A tail, and the polymerases involved in Eberwine-type amplification/labeling steps must complete their processes over this entire distance. Therefore, any variation in mRNA integrity or in polymerase processivity should affect the measured intensity by a factor proportional to the number of bases between the probe target and the 3' poly-A tail. Also, variable amounts of starting mRNA could affect the final diversity of the amplified cRNA, such that a sample with less starting mRNA would tend to have fewer reliably measured genes and, therefore, a higher signal-to-noise ratio.

In our current analysis we hypothesized that a set of four metrics could characterize the relative amount of bias affecting each sample in a data set. To capture the saturation and noise floor of the raw probe intensities, we used the median and interquartile range (IQR) of the perfect-match probes on the array ('PM median' and 'PM IQR'). The effect of RNA degradation was estimated by the average decrease in expression between 5' probes and 3' probes ('degradation'). Finally, the diversity of starting mRNA was characterized by the IQR of the RMA-summarized expression values ('RMA IQR').

In the Signoretti data set, each of the four bias metrics is correlated with the expression values of more probe sets than expected by chance (Figure 3). When applied to the gene expression vectors in multivariate linear regression, the four bias metrics explained 33% of the variance, whereas a set of random vectors would be expected to explain 4%. The correlation between probe sets and the bias metrics was strongly intensity-dependent and, thus, may be the source of the intensity-dependent correlation bias (Figure 4). The RMA IQR and degradation bias metrics together explain 44% of the variance in the sample distance matrix, indicating that the dendrogram derived from all genes in Figure 1a was largely driven by technical bias.

**Figure 3:**
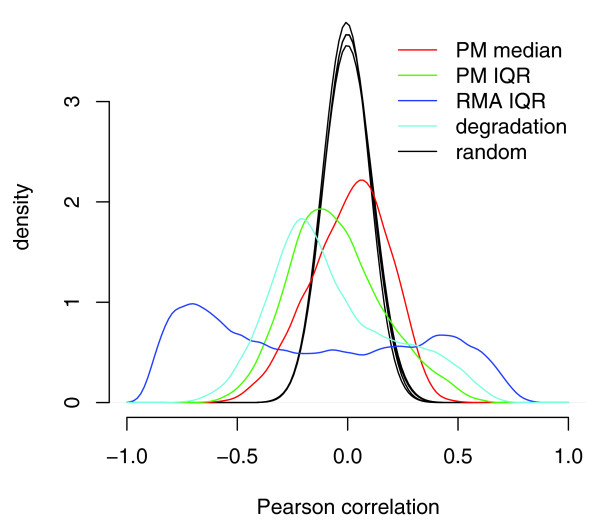
The expression values of many probe sets are correlated with each of four bias metrics. For each bias metric, a density curve indicates the distribution of Pearson correlation coefficients between the bias metric and the expression values of each probe set. The four black curves (which appear as one) indicate the distribution of correlation coefficients between the expression values of each probe set and 100 random permutations of each bias metric.

**Figure 4:**
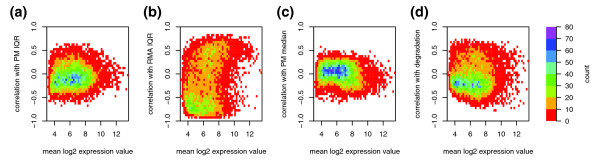
The correlation between genes and bias metrics is non-randomly distributed according to mean gene intensity. For each probe set in the breast cancer data set, we calculated the mean intensity (across all 98 samples) and the correlation with a bias metric (also across all 98 samples). The values for all 12,625 probe sets were summarized as two-dimensional histograms for each of the four bias metrics: **(a) **PM IQR; **(b) **RMA IQR; **(c) **PM median; and **(d) **degradation.

Since the bias metrics did not correspond to any available clinical covariates (hormone receptor status, HER-2 amplification, tumor grade), we removed the component of the expression matrix explained by the bias vectors (see Materials and methods). The resulting bias-corrected data had substantially less intensity-dependent correlation bias (Figure 2b), and when we subjected it to hierarchical clustering we found that all nine replicates clustered together (Figure 1c). In a separate glioma data set with two pairs of replicates [17], an identical procedure also increased the number of pairs clustering together (Figure 5). Thus, this bias correction method seems to improve the correspondence between hierarchical clusters and known relationships between samples without filtering out the majority of the genes.

**Figure 5:**
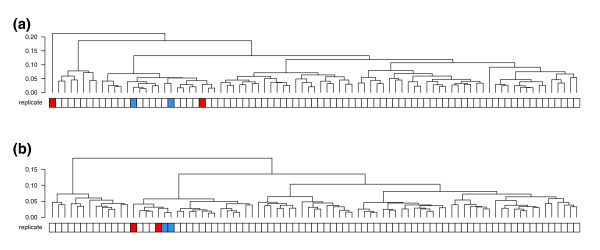
Bias correction improves concordance of replicates in a glioma gene expression study. Hierarchical clustering was performed as in Figure 1. **(a) **The unfiltered, uncorrected data results in neither of the two replicate pairs together. **(b) **Bias correction as described in this paper results in one of two replicate pairs clustered together.

To investigate the effects of bias correction on single-gene analysis, we used the estrogen receptor (ER) status of breast tumors as a reference point [7]. Since the ER-positive and ER-negative subtypes are observed in many data sets and on several microarray platforms, the correlation between ER status and the expression level of any given gene should be relatively well conserved between data sets of sufficient size. The correlation of correlations (CC) is a measure of consistency in gene-phenotype association between one data set and another [18]. We calculated the pairwise CCs between five breast cancer data sets with a large number of patients (*n *range 99-289), a common platform (Affymetrix HG-U133A), and ER status annotation [8-10,12,19]. Bias removal increased the average CC from 0.70 to 0.76, although not all pairs of data sets were improved (Figure 6a). Notably, it was the data set pairs with an initially low CC that benefited most from bias correction. In the most extreme case the CC between two data sets increased from 0.55 to 0.76 after bias correction (Figure 6b, c).

**Figure 6:**
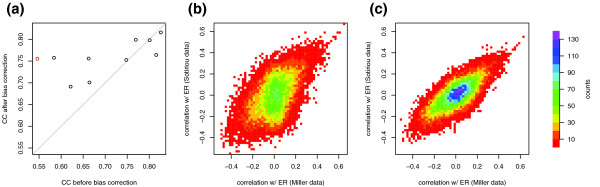
Bias correction increases consistency of correlation with ER status within five clinical breast cancer studies. **(a) **CC coefficients are plotted for each possible pair of data sets, before and after bias correction. The largest improvement, indicated by a red circle, is between data sets from Sotiriou *et al*. [19] and Miller *et al*. [9]. For these two data sets, two-dimensional histograms indicate the distribution of correlation coefficients between expression values and ER status for all probe sets **(b) **using uncorrected data and **(c) **after bias correction.

As an alternative to our bias correction method, we considered batch adjustment, in which the expression matrix is adjusted, one probe set at a time, to remove any difference in means between the batches. From the scan dates embedded in the CEL files, we inferred that the Signoretti breast cancer data were scanned in three batches. We applied batch adjustment to this data set and found that the number of correctly paired replicates increased from four to five (data not shown). Thus, on this data set, batch adjustment is less effective than our bias correction method. The other data sets used in this study were each scanned in a relatively large number of batches (median, 14; range, 8-31), so batch adjustment was not attempted.

## Conclusion

Various analytical approaches are susceptible to errors caused by technical bias. For simpler analyses of individual genes (for example, detection of differentially expressed genes between previously defined groups), technical bias may manifest as an unmodeled factor that complicates significance testing [20]. However, if a bias metric is correlated with a biological or clinical variable of interest, separating bias effects from biologically relevant effects may be difficult. A strong correlation may indicate that the groups of samples were collected or processed under inconsistent conditions, a situation that should be avoided [21].

More complex analyses have inferred functional relationships between genes based on their coordinated expression [22]. Such approaches may be especially sensitive to technical bias, because the coordinated effect of technical bias on multiple probe sets causes spurious correlations between these probe sets. On the other hand, coordinated bias on multiple genes also affects correlation between samples, such that two samples with dissimilar levels of bias may appear to have very different expression profiles. We have demonstrated that this bias affects sample clustering, but other visualization methods, such as principal components analysis, can be similarly distorted (data not shown). Similarly, recent work has shown that cellular network reconstruction from gene expression data can be strongly influenced by artifacts introduced during normalization [23].

We have found that technical bias can substantially affect clinical microarray data, and our results indicate that it should be corrected, or at least considered as a potential confounding effect, in any analysis. We have presented a simple approach for correction of biased data, but it may not be optimal for all data sets. Our choice of bias metrics was based on consideration of the processes involved in the generation of microarray data, but other variables, such as those used to justify the exclusion of outlying arrays, may also quantify technical bias [24]. As more clinical data sets with known biological relationships (for example, replicates) become available, it will be possible to explore more sophisticated models for bias correction.

## Materials and methods

All analysis was performed using the R statistical environment [25], with use of the 'affy' package from Bioconductor [26]. Each data set was normalized/summarized individually using RMA [6], unless otherwise noted. All correlations are Pearson product-moment correlations. Hierarchical clustering was performed using complete linkage and Pearson correlation distance. An R package implementing our method, as well as raw data and scripts to reproduce our results, are available on our website [27].

### Post-summary quantile normalization

A reference distribution was calculated by averaging the sorted expression values across all samples. Then, for each sample, the original expression values were replaced by those of the reference distribution in the same order, with ties broken at random. This results in all samples in a data set having exactly the same distribution of expression values.

### Intensity-dependent correlation bias

First, the *n *rows (probe sets) of the expression matrix were sorted according to mean expression value. The rows were then split into 50 bins of approximately equal size. Thus, the first bin contains the *n*/50 probe sets having the lowest mean signal, and the last bin contains the *n*/50 probe sets having the highest mean signal. For each possible pair of bins, all (*n*/50) × (*n*/50) pairwise Pearson correlation coefficients (between rows) were calculated, and the median correlation coefficient was recorded. These median correlation coefficients for each pair of bins were plotted as a color-coded 50 × 50 matrix. Note that for the diagonals of the color-coded matrix, which corresponds to the correlation between a bin and itself, only correlations between non-identical probe sets were counted.

### Bias metrics

The four bias metrics defined here were calculated for each microarray sample: PM Median isthe median of the log_2_-transformed perfect-match probe intensities; PM IQR is the interquartile range of the log_2_-transformed perfect-match probe intensities; RMA IQR is the interquartile range of all summarized RMA values (which are already log_2_-transformed); and 'degradation' is the slope of the least-squares regression of the log_2_-transformed perfect-match probe intensities versus their relative position (within the probe set) along the targeted transcript - this was calculated with the AffyRNAdeg function in the Bioconductor package affy [26].

### Correlation between bias metrics and expression values

To calculate the random distributions in Figure 3, each bias metric was randomly permuted 100 times. We computed Pearson correlation coefficients between each permutation and expression values of each of the 12,625 probe sets; thus, each density curve represents 100 × 12,625 correlation coefficients. This procedure was repeated for each of the four bias metrics.

### Bias correction

Let *E*_*ij *_be the RMA-normalized expression value of probe set *i *in sample *j*. The four bias metrics *B*_*mj *_(*m *∈ 1..4) were calculated for each sample *j *independently. The effect of technical bias on the expression levels was estimated by least-squares regression with the bias metrics as independent variables. Specifically, for each probe set *i *independently, the parameters *α*_*i *_and *β*_*im *_were chosen to minimize the sum square of the residuals *ε*_*ij*_.

$$E_{ij}=\alpha_{i}+\sum_{m=1}^{4}\beta_{im}B_{mj}+\varepsilon_{ij}$$

The bias-corrected expression values ${E^{\prime }}_{ij}$ are equal to the residuals plus an offset (to preserve the mean expression for each probe set):

$${E^{\prime }}_{ij}=\varepsilon_{ij}+{\bar{E}}_{i\cdot }$$

### Correlation-of-correlations

For each data set, we calculated the Pearson correlation coefficient of each probe set with the binary ER status. The CC is the Pearson correlation coefficient of the resulting vector with the corresponding vector from a different data set. Thus, the CC indicates how well two data sets agree on the extent to which each probe set is correlated with the ER status.

## Abbreviations

CC, correlation of correlations; ER, estrogen receptor; GCRMA, GeneChip robust multi-array average; IQR, interquartile range; MAS5, Microarray Suite 5; MBEI, model-based expression index; PSQN, post-summary quantile normalization; RMA, robust multi-array average.

## Authors' contributions

ACE and ZS conceived and designed the study. ACE performed all analysis. ZS provided guidance. Both authors wrote, read, and approved the final manuscript.

## Additional data files

The following additional data are available with the online version of this paper. Additional data file 1 is a figure showing that intensity-dependent correlation bias is present in all data sets tested.

## Supplementary Material

Additional data file 1 Eight arbitrarily chosen cancer data sets [8-12] were tested for intensity-dependent correlation bias. For each data set, the expression values were calculated from the raw data using the RMA, MAS5, MBEI, and GCRMA normalization algorithms. Regardless of the normalization algorithm, intensity-dependent correlation bias was present, and PSQN reduced this bias. Click here for file
