# Supplementary figures and images for: Correction of technical bias in clinical microarray data improves concordance with known biological information
Source: Genome Biol. 2008 Feb 4;9(2):R26. doi: 10.1186/gb-2008-9-2-r26 (PMC2374720; doi:10.1186/gb-2008-9-2-r26)

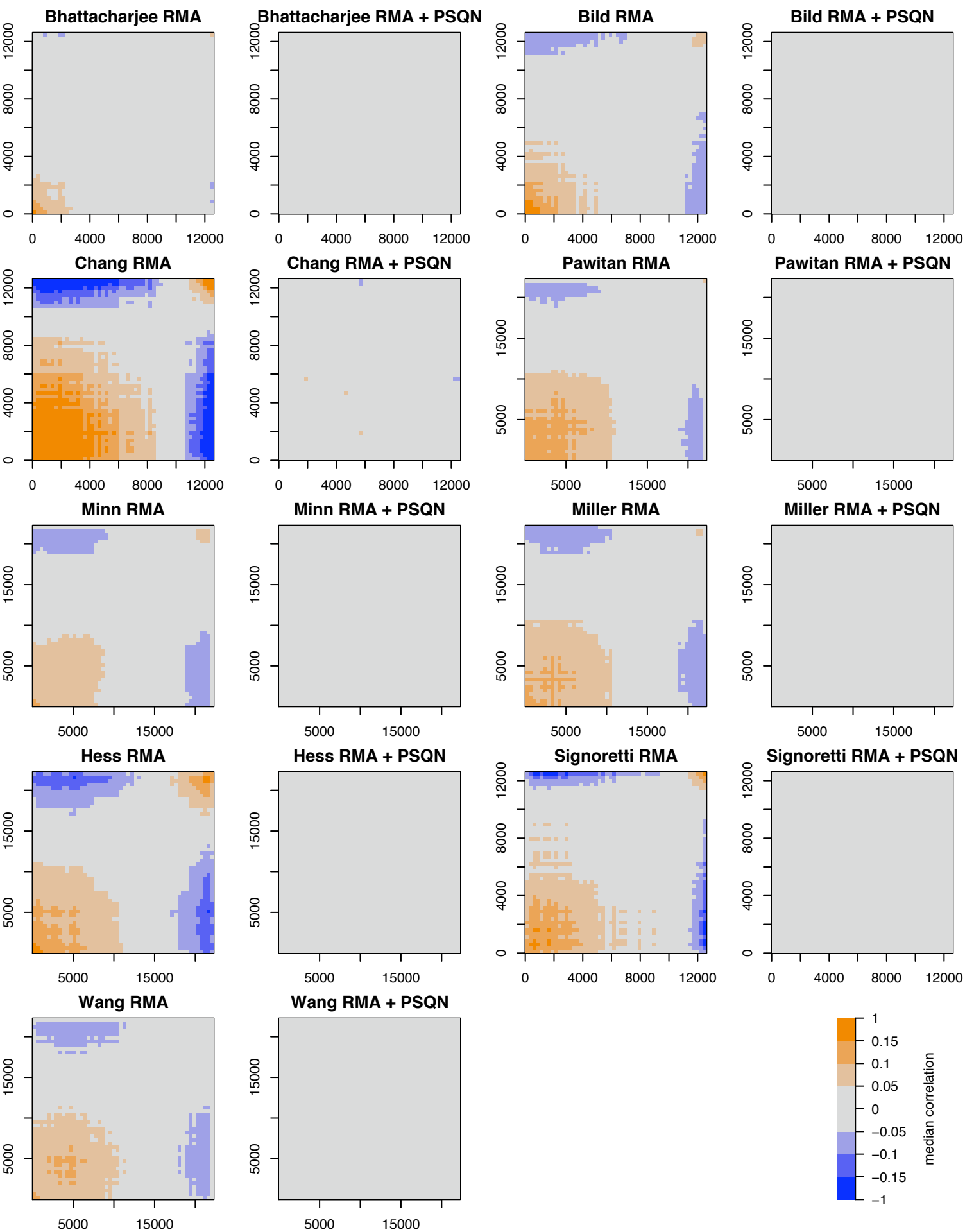

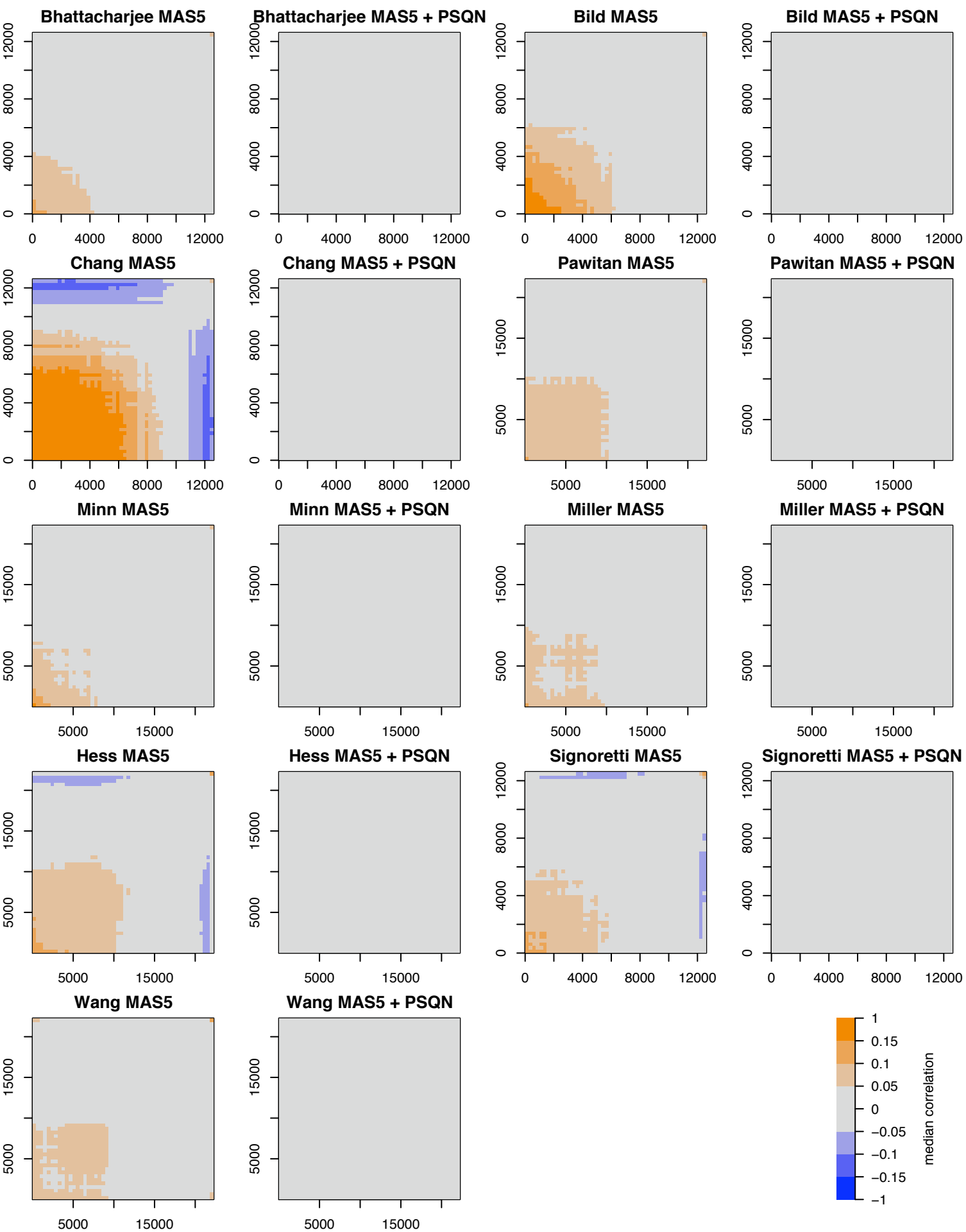

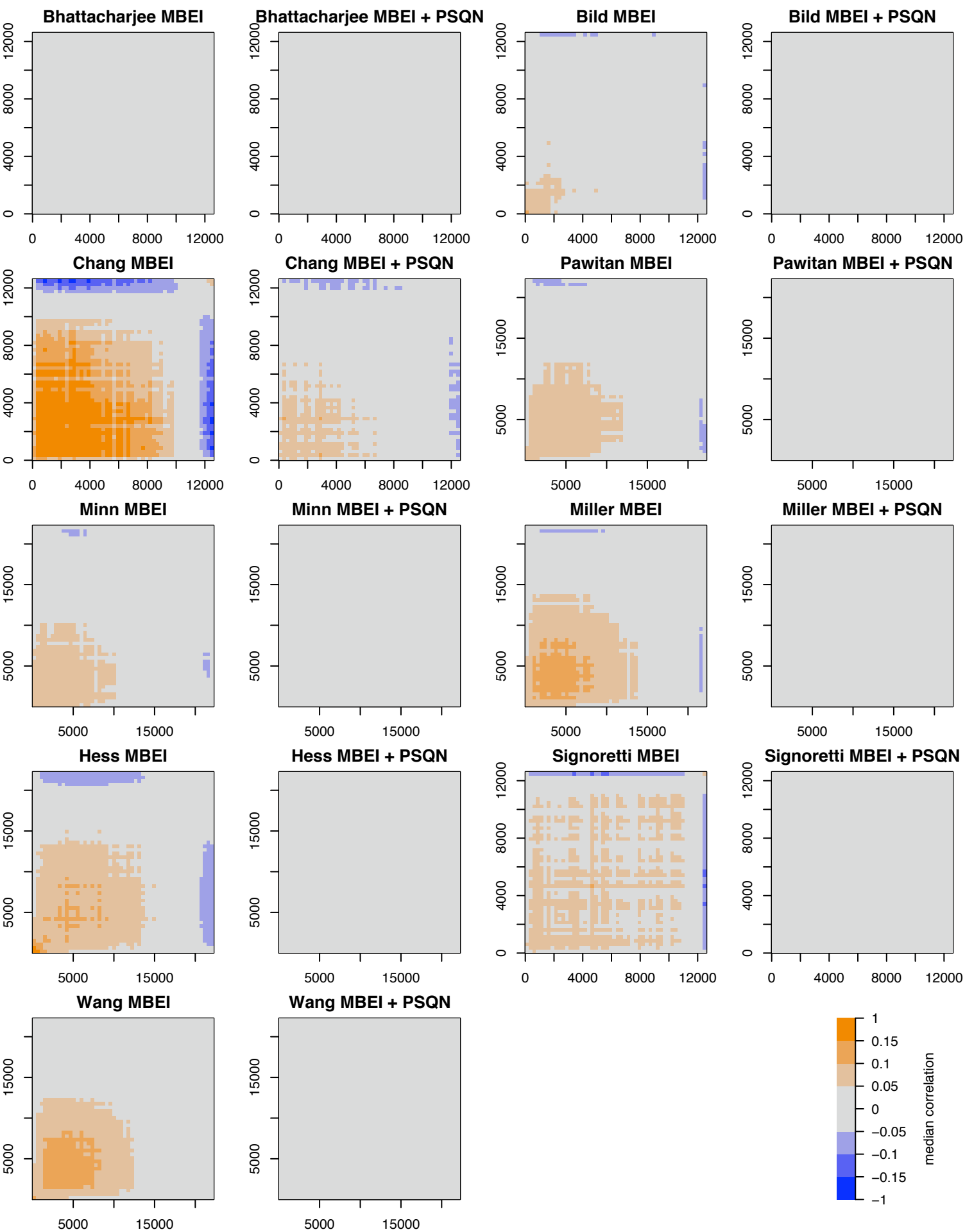

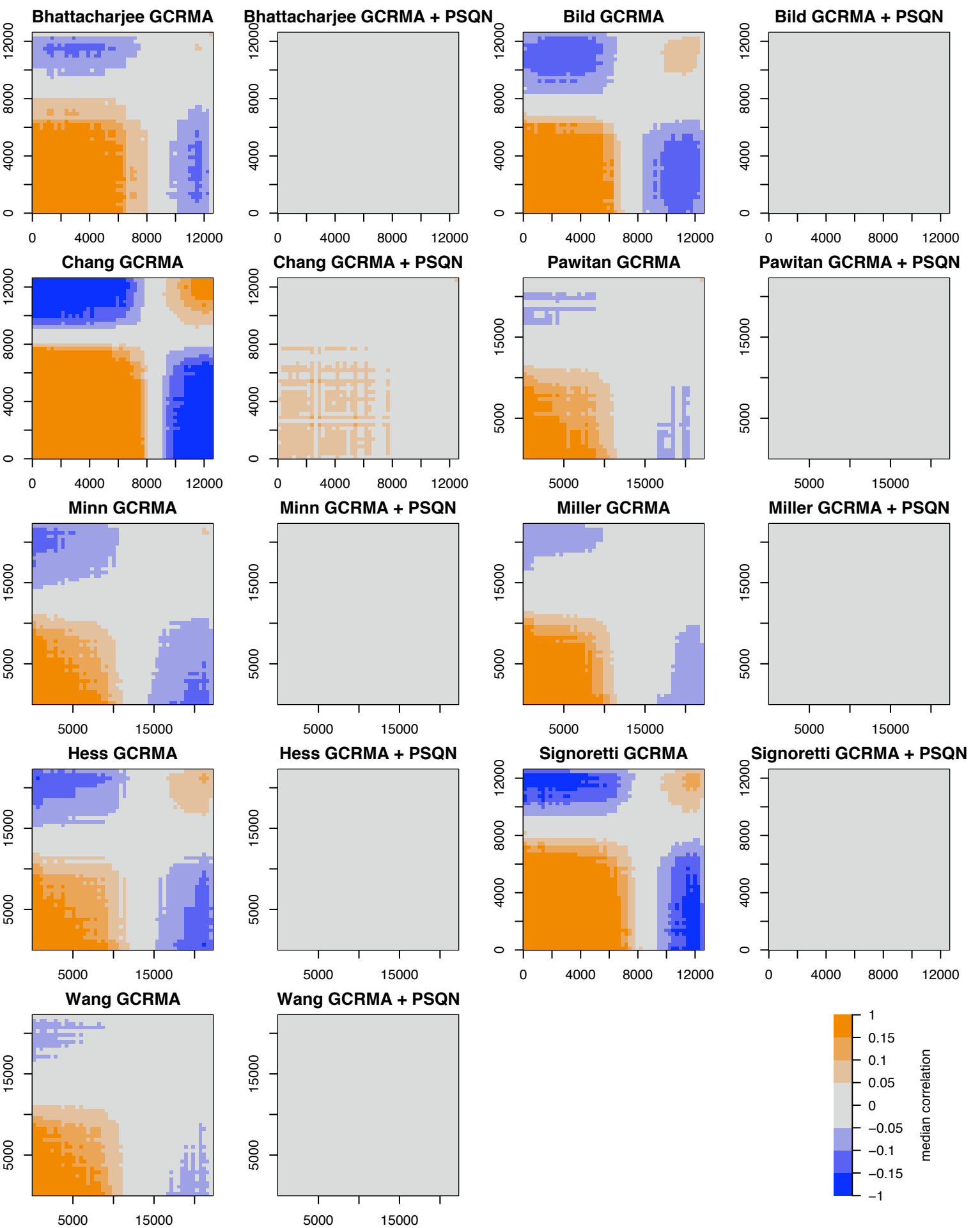

Supplement: Additional data file 1 — Eight arbitrarily chosen cancer data sets [8-12] were tested for intensity-dependent correlation bias. For each data set, the expression values were calculated from the raw data using the RMA, MAS5, MBEI, and GCRMA normalization algorithms. Regardless of the normalization algorithm, intensity-dependent correlation bias was present, and PSQN reduced this bias. [file gb-2008-9-2-r26-S1.pdf]
